# Mechanisms of Nrf2/Keap1-Dependent Phase II Cytoprotective and Detoxifying Gene Expression and Potential Cellular Targets of Chemopreventive Isothiocyanates

**DOI:** 10.1155/2013/839409

**Published:** 2013-05-28

**Authors:** Biswa Nath Das, Young-Woo Kim, Young-Sam Keum

**Affiliations:** College of Pharmacy, Dongguk University, 813-4 Siksa-dong, Goyang, Gyeonggi-do 410-820, Republic of Korea

## Abstract

Isothiocyanates (ITCs) are abundantly found in cruciferous vegetables. Epidemiological studies suggest that chronic consumption of cruciferous vegetables can lower the overall risk of cancer. Natural ITCs are key chemopreventive ingredients of cruciferous vegetables, and one of the prime chemopreventive mechanisms of natural isothiocyanates is the induction of Nrf2/ARE-dependent gene expression that plays a critical role in cellular defense against electrophiles and reactive oxygen species. In the present review, we first discuss the underlying mechanisms how natural ITCs affect the intracellular signaling kinase cascades to regulate the Keap1/Nrf2 activities, thereby inducing phase II cytoprotective and detoxifying enzymes. We also discuss the potential cellular protein targets to which natural ITCs are directly conjugated and how these events aid in the chemopreventive effects of natural ITCs. Finally, we discuss the posttranslational modifications of Keap1 and nucleocytoplasmic trafficking of Nrf2 in response to electrophiles and oxidants.

## 1. Regulation of Nrf2-Dependent Gene Expression by Natural Isothiocyanates

**Figure 1:**
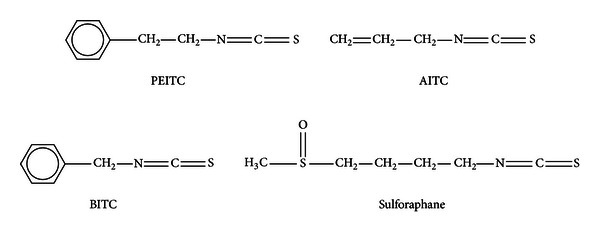
Chemical structure of selected natural isothiocyanates (ITCs).

Natural Isothiocyanates (ITCs) are abundantly found in cruciferous vegetables such as broccoli, watercress, Brussels sprouts, cabbage, and cauliflower [1]. Epidemiological studies have shown that consumption of cruciferous vegetables is inversely associated with the risk of many types of cancer [2]. Anticarcinogenic properties of cruciferous vegetables might be attributed to their high content of glucosinolates and the composition of the glucosinolates among cruciferous vegetables differs, depending on the plant species, climates, and other agricultural conditions [3]. Glucosinolates in cruciferous vegetables exist as *N*-hydroxysulfate with sulfur-linked *β*-glucose together with various side chains [4]. Naturally occurring glucosinolates are converted into isothiocyanates (ITCs) with a physical stress, such as chopping or chewing of cruciferous vegetables, which in turn leads the plant cell wall to rupture and release the plant-specific enzyme myrosinase, converting the natural glucosinolates into ITCs [5]. Naturally occurring ITCs, including phenethyl ITC (PEITC), allyl ITC (AITC), benzyl ITC (BITC), and sulforaphane are effective cancer chemopreventive compounds in humans (Figure 1) [6]. While many dietary chemopreventive compounds (e.g., curcumin, resveratrol, and epigallocatechin gallate (EGCG)) possess polyphenolic moiety, chemopreventive ITCs are structurally distinct in that they are characterized by –N=C=S functional group [7].

The anticarcinogenic mechanisms of ITCs include a variety of biochemical mechanisms, such as cell cycle arrest, apoptosis induction, activation of anti-inflammatory programs, inhibition of cytochrome P450s for carcinogen activation, and modulation of the activities of various transcriptional factors, including NF-E2-related factor 2 (Nrf2) [8]. Nrf2 is a member of cap^“^n^”^collar (CNC) family of basic leucine zipper (bZIP) transcription factor that allows cells to mediate a collective activation of phase II cytoprotective and detoxifying enzymes [9]. Phase II cytoprotective and detoxifying enzymes are implicated in the generation of cellular reduced glutathione (GSH), detoxification of reactive oxygen species (ROS), and drug metabolism in response to environmental electrophiles and oxidants [10]. Under normal condition, Nrf2 is constantly polyubiquitinated and degraded by proteasome through Cullin-3- (Cul3-) dependent E3 ubiquitin ligase enzyme. Exposure of electrophiles and oxidants inactivates Cul3-dependent E3 ubiquitin ligase enzyme in the cytosol by poorly characterized biochemical mechanisms and stabilizes Nrf2 protein, leading to its nuclear translocation and transcriptional activation by binding to the antioxidant response element (ARE), a *cis*-acting enhancer sequence TGA(G/C)NNNGC in the genome through heterodimerizing with small Maf proteins [11]. Nrf2 activity is tightly regulated in the cytosol by Kelch-like ECH associating protein 1 (Keap1) as a scaffolding protein for Nrf2 as well as an adaptor protein for Cul3-dependent E3 ubiquitin ligase enzyme [12]. Analysis of Keap1-deficient mice has proven that Keap1 plays a central role in the repression of Nrf2 activity *in vivo* [13]. In addition, loss of Keap1 activity as a result of somatic mutations has been reported in a significant proportion of cancer patients, implying that constitutive activation of Nrf2 may have an important role in the elevated cytoprotective activity of human malignancy [14].

**Figure 2:**
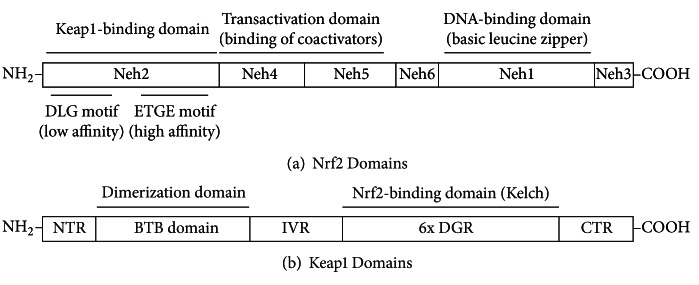
Nrf2 and Keap1 protein domains.

Nrf2 possesses 6 conserved Nrf2-ECH homology (Neh) domains (Figure 2(a)). The Neh1 domain contains a basic leucine-zipper (bZIP) structure, required for DNA binding in association with small Maf proteins in the nucleus. The Neh2 domain is located in the most N-terminal region and exerts a negative effect on the ARE-dependent gene expression by binding to Keap1 protein. The Neh4 and Neh5 domains constitute transactivation domains that contribute to ARE-dependent gene activation by binding to coactivators, such as CBP and p300, and are essential for Nrf2 transactivation [15]. The Neh3 domain, located in the most C-terminal region, is known to play a permissive role in Nrf2 transactivation for the Neh4 and Neh5 domains. The Neh6 domain, located between the transactivation domain (the Neh4 and Neh5 domains) and the DNA binding domain (the Neh1 domain), is known to be necessary for the degradation of Nrf2 protein [16]. Keap1 is a negative regulator of Nrf2 protein by binding to the Neh2 domain of Nrf2 and was initially identified by a yeast two-hybrid assay [17]. Keap1 protein is a cytosolic protein and comprises 5 different domains: an amino-terminal region (NTR), a Broad complex, Tramtrack and Bric a bric (BTB) domain, an intervening region (IVR), six Kelch/double glycine repeats (DGRs), and a carboxy-terminal region (CTR) (Figure 2(b)) [18]. Structural analysis has shown that Keap1 proteins heterodimerize each other through the BTB domain, and the overall heterodimers resemble a “cherry-bob” structure [19]. Covalent modification of cysteine residues in Keap1 protein is believed to constitute a stress-sensing mechanism for electrophiles and oxidants, and the covalent binding of several electrophiles and thiol group(s) in Keap1 protein has been observed *in vitro*, including sulforaphane [20]. Structural observations and biophysical experiments have led to the conclusion that (1) the ratio between Keap1 and Nrf2 binding is 2 : 1 and (2) the regulatory mechanism of Nrf2 and Keap1 system conforms to the so-called “hinge and latch” model, in which two distinct binding sites in the Neh2 domain of Nrf2 protein mediates high-affinity (the ETGE motif) and low-affinity (the DLG motif) interactions with a single Keap1 protein, respectively [21].

## 2. Indirect and Direct Protein Targets of Natural ITCs

Until now, the exact biochemical mechanisms by which ITCs activate Nrf2-dependent gene expression are largely unclear. However, there is an increasing number of evidence, showing that ARE-dependent transcriptional gene activation by ITCs is mediated, at least in part, by the activation of various intracellular signaling cascades, including the mitogen-activated protein kinase (MAPK) [22]. MAPK is one of the major signaling systems, which transmits various extracellular signals into the nucleus through a cascade of serial intracellular protein phosphorylation and is known to be responsible for the activation of ARE-dependent gene expression [23]. MAPK consists of three family members: extracellular signal-related kinase (ERK), c-jun N-terminal kinase (JNK), and p38 MAPK. MAPK is phosphorylated and activated by upstream signaling kinase modules, for example, MAPK kinase (MAPKK or MEK) and MAPKK kinase (MAPKKK or MEKK). Upon activation, MAPK is phosphorylated in both threonine (T) and tyrosine (Y), existing in the TXY motif of activation loop and the central amino acid (X) is a defining amino acid motif for individual MAPKs: glutamic acid (E) for ERK, proline (P) for JNK, and glycine (G) for p38 MAPK [24]. Earlier studies have demonstrated that overexpression of wild-type ERK2 and JNK1 significantly elicited ARE-dependent luciferase activation and the addition of natural ITCs, including PEITC and sulforaphane, could potentiate the ARE-dependent gene expression, implying that upregulation of Nrf2/ARE-dependent gene expression by natural ITCs is mediated by MAPK pathway [25, 26]. While the positive regulation of Nrf2/ARE-dependent gene expression by ERK and JNK has been unequivocally supported by follow-up studies [27, 28], the exact role of p38 MAPK pathway in the ARE-dependent gene expression is still controversial, although a direct binding and phosphorylation residue(s) of Nrf2 protein by p38 MAPK has been demonstrated [29, 30]. In addition, the experimental evidence showing the direct phosphorylation and the exact residue(s) of Nrf2 or Keap1 protein by activated MAPK is still lacking. Therefore, it seems likely that the modulation of Nrf2/ARE-dependent gene expression by MAPKs is indirect. 

Phosphatidylinositol 3-kinase (PI3K) is another intracellular signaling kinase that is implicated in the regulation of Nrf2/ARE-dependent gene expression. Earlier studies have demonstrated that PI3K and its downstream Ser/Thr kinase, Akt can positively regulate ARE-dependent gene expression. While there was a lack of evidence whether PI3K and Akt can directly phosphorylate Keap1 or Nrf2 protein and modulate the activity of ARE-dependent gene expression, Cuadrado and colleagues have demonstrated that active glycogen synthase kinase-3*β* (GSK3*β*) can directly phosphorylate and suppress the activity of Nrf2 protein by causing its nuclear exclusion [31]. GSK3*β*, a direct downstream target of Akt, is activated in response to growth factors and external oxidants such as H_2_O_2_ [32]. Because GSK3*β* activity is negatively regulated by Akt-mediated phosphorylation at Ser-9, it is possible to assume that PI3K-mediated Akt activation might cause a phosphorylation and inactivation of GSK3*β*, thereby promoting Nrf2 nuclear translocation and activation by relieving GSK3*β*-mediated negative regulation of Nrf2 activity. In addition, a novel phosphodegron motif, existing in the Neh6 domain of Nrf2 (DSGIS residues 334 to 338) was identified in the subsequent study, in which Nrf2 protein is destabilized as a consequence of its phosphorylation by GSK3*β* and subsequent recognition and polyubiquitination by Cul1/Skp1/*β*-TrCP E3 ubiquitin ligase enzyme, but not by Cul3/Keap1 E3 ubiquitin ligase enzyme [33]. In addition, Jaiswal and colleagues have identified that Fyn kinase can directly phosphorylate Nrf2 protein at Tyr-568 and promote its nuclear exclusion and degradation, thereby contributing to the suppression of ARE-mediated gene expression [34]. They also showed that GSK3*β* acts as an upstream kinase of Fyn that contributes to phosphorylation of Nrf2 protein at Tyr-568 [35]. Therefore, it seems likely that the PI3K-Akt-GSK3*β* axis regulates Nrf2-mediated ARE-dependent gene activation both in direct and indirect manners: GSK3*β* directly phosphorylates the phosphodegron motif existing in the Neh6 domain of Nrf2 protein and it leads to Keap1-independent, but *β*-TrCP-dependent proteasomal degradation of Nrf2 protein or GSK3*β* phosphorylates and activates Fyn kinase, leading to phosphorylation and an indirect nuclear exclusion of Nrf2 protein. At present, whether and, if it is so, how natural ITCs modulate GSK3*β* or Fyn kinases to Nrf2-dependent ARE activation is currently unknown. In addition to MAPK and PI3K/Akt/GSK3*β*/Fyn cascades, protein kinase C (PKC) and PKR-like endoplasmic reticulum kinase (PERK) are the other intracellular kinases to directly phosphorylate Nrf2 protein and modulate ARE-dependent gene expression. PKC directly phosphorylates Nrf2 protein at Ser-40 [36] and upregulates the Nrf2-mediated ARE activation by perturbing the interaction between Nrf2 and Keap1 proteins [37]. PERK can directly phosphorylate Nrf2 protein following the accumulation of unfolded proteins of endoplasmic reticulum, although the exact phosphorylation residue(s) were unidentified [38]. While it is largely unclear how Nrf2 phosphorylation contributes to ARE-dependent gene expression, Apopa et al. have provided interesting results, showing that treatment of *tert*-butylhydroquinone (tBHQ) elicited casein kinase 2- (CK2-) mediated phosphorylation of Nrf2 protein, thereby facilitating its nuclear translocation and activation of ARE-dependent gene expression [39]. This fact implies that Nrf2 phosphorylation might be closely associated, at least in part, with the nucleocytoplasmic trafficking of Nrf2 in cells.

As mentioned earlier, the chemopreventive mechanisms of ITCs are diverse and it is likely due to the fact that ITCs readily react with the nucleophilic amino acid residues. Based on this conjecture, Chung and colleagues have attempted to find out whether ITCs can directly react with cellular DNA, RNA, and proteins. To this end, they have exposed ^14^C-PEITC and ^14^C-sulforaphane in cultured cells and purified nucleotides or target proteins, using phenol/chloroform extraction or two-dimensional electrophoresis (2D-GE) followed by matrix-assisted laser desorption-ionization mass-spectrometry (MALDI-MS) [40]. As a result, they found that no discernable DNA or RNA was bound to radiolabeled ITCs, suggesting that nucleotides are unlikely direct targets for ITCs [41]. In contrast, several putative protein targets to which ITCs can be directly conjugated were identified. They include cellular reduced glutathione (GSH), tubulin, transient receptor potential channel, phosphatases (M3/6 and cdc25c), MEKK1 kinase, and transcriptional factors, such as activator protein-1 (AP-1), signal transducer and activator of transcription factor 3 (STAT3), and mutant p53 [42]. It is known that ITCs can be directly conjugated to thiol group-containing cysteine, amine group-containing lysines, arginines, proline, serines, threonine, and tyrosine. Among them are cysteines wich are the most likely binding sites for ITCs and cysteine residues in the above-mentioned proteins are the possible conjugation candidates [43]. In addition, finding out the direct binding proteins for ITCs has been attempted in an alternative manner by taking advantage of affinity chromatography technique. To this end, HeLa cell lysates were incubated with biotin-labeled ITCs, separated with streptavidin-sepharose beads, and sent for mass spectrometry analysis. This approach was useful in revealing the direct conjugation of ITCs with a number of novel proteins, including macrophage-inhibitory factor (MIF) [44]. However, this approach has its weakness in that ITCs are strong electrophiles, and a false-positive binding of ITCs with nontarget protein(s) might likely occur. 

## 3. Direct or Indirect Modulation of Keap1/Nrf2 Proteins by ITCs

ITCs are strong chemical inducers of ARE-dependent gene expression. Therefore, it is possible to assume that ITCs might be able to induce ARE-dependent gene expression by altering the interaction between Keap1 and Nrf2 proteins through a direct conjugation with cysteine residues in Keap1 or Nrf2 protein. In particular, Keap1 is a cysteine-rich protein (27 for human and 25 for mouse) with 4.3% of all residues being cysteines that exceed the average percentage of cysteine residues in proteins [45]. Because cysteines generally constitute the functional and redox-sensitive domains of proteins in response to the changes in the local environment [46], cysteine residues in Keap1 protein were proposed to be the prime mechanism, by which they selectively respond to a variety of electrophiles and oxidants. This hypothesis was supported by the observation that universal ARE inducers can react with cysteine sulfhydryl groups of Keap1 at rates that correlated with their potency of ARE-dependent gene activation, irrespective of their chemical structures [47]. It was shown that reactive cysteines were mostly located in the linker region, located between the BTB domain and the Kelch-repeats in Keap1 protein. The selective modification of cysteine residues in Keap1 protein by structurally similar Nrf2 chemical inducers led to the so-called hypothesis “cysteine code” or “multiple-sensor mechanism” [48]. Unlike Keap1, however, Nrf2 protein was excluded as a sensor for electrophiles or oxidant in this model because it contained no cysteines in the Neh2 domain. Nonetheless, it should be noted that observing a direct binding between Nrf2 chemical inducers and Keap1 protein was made in the test tube, using a recombinant protein. In addition, the experimental evidence that natural ITCs could be directly conjugated to any of cysteine residues in cellular Keap1 and Nrf2 proteins is still lacking. In this sense, Takaya et al. have recently observed that a point mutation of cysteine 151 resulted in a reduced Nrf2 activation in response to several Nrf2 inducers, including sulforaphane, but not to other inducers such as CDDO-Im and cadmium chloride [49]. This fact suggests a potential role for cysteine 151 of Nrf2 protein in sulforaphane-mediated ARE activation, although it is unclear yet whether this residue serves as a direct binding site for sulforaphane.

By now, significant attention has been focused on the modification of cysteine residues in Keap1. However, it is also possible to envisage that Nrf2 cysteine modification can serve as another potential mechanism for ARE-dependent gene regulation. To this end, He and Ma have demonstrated that selected evolutionary conserved cysteine residues in Nrf2 can be directly modified by arsenic or phenylarsine oxide (PAO), and these residues are important for its binding to ARE-dependent gene expression. This raises an interesting possibility that direct modification of Nrf2 amino acid residue(s) by ARE inducers constitutes an alternative mechanism for ARE activation [50]. In another study, Li et al. have identified a potential nuclear export sequence (NES) motif in the Neh5 transactivation domain of Nrf2 protein and observed that mutating cysteine residue at 183 position into alanine (C183A) abrogated the NES function of Nrf2, rendering a nuclear accumulation of Nrf2 [51]. In addition, several potential NES sequence motifs together with putative nuclear localization sequence (NLS) motifs were identified in the Nrf2 protein sequence [52]. Interestingly, Li et al. showed that EGFP-tagged Nrf2 segment (amino acids 162–295), in which a putative NES exists exhibited a cytosolic pattern and that an exposure of oxidants or electrophiles, including sulforaphane could alter subcellular localization of EGFP-tagged Nrf2 segment. This result suggests that this NES sequence is redox sensitive [51]. In contrast, they have conducted analogous experiments and demonstrated that some NES/NLS motifs might be redox insensitive; the subcellular localization of these NES/NLS is unaltered by treatment of many electrophiles and oxidants [53]. Collectively, these studies show that multiple NES/NLS motifs play an important role in the nucleocytoplasmic localization of Nrf2 protein and suggest that Nrf2 protein by itself might be able to behave as a Keap1-independent sensor. However, it is still uncertain whether these residues are direct targets of natural ITCs. Therefore, whether these cellular cysteine residues in Keap1 and/or Nrf2 serve as direct targets of ITCs requires experimental validations. More importantly, whether and, if so, how cysteine modifications of Keap1 and Nrf2 by ITCs are linked to phosphorylation-mediated regulation of Keap1 and/or Nrf2 activities needs to be further clarified.

## 4. Concluding Remark

To adapt to their aerobic lifestyle, mammals have developed an elaborate *in vivo* defense and metabolizing enzyme system. As mentioned earlier, Keap1/Nrf2-regulated gene expression of phase II cytoprotective and detoxifying enzymes is one of such prime cytoprotective mechanisms, and we are already aware that natural ITCs exploit this pathway to exert chemopreventive effects in humans. In addition, we have provided an overview of current knowledge regarding the direct and/or indirect cellular targets for ITCs. As mentioned earlier, whether cellular Nrf2 and/or Keap1 proteins are direct targets of ITCs is currently unknown, and there is a great deal of research conducted to fill this knowledge gaps, to the best of our knowledge. Recent analysis of Nrf2 interactome and regulome also highlights an enormous array of potential targets of natural ITCs and suggests that chemopreventive mechanisms, exerted by chemopreventive ITCs, might be much more complex than initially imagined [54].

## References

[1] Zhang Y (2012). The molecular basis that unifies the metabolism, cellular uptake and chemopreventive activities of dietary isothiocyanates. *Carcinogenesis*.

[2] Zhang Y (2004). Cancer-preventive isothiocyanates: measurement of human exposure and mechanism of action. *Mutation Research*.

[3] Fahey JW, Zalcmann AT, Talalay P (2001). The chemical diversity and distribution of glucosinolates and isothiocyanates among plants. *Phytochemistry*.

[4] Zhang Y, Li J, Tang L (2005). Cancer-preventive isothiocyanates: dichotomous modulators of oxidative stress. *Free Radical Biology and Medicine*.

[5] Conaway CC, Yang YM, Chung FL (2002). Isothiocyanates as cancer chemopreventive agents: their biological activities and metabolism in rodents and humans. *Current Drug Metabolism*.

[6] Keum YS, Jeong WS, Kong AN (2005). Chemopreventive functions of isothiocyanates. *Drug News and Perspectives*.

[7] Keum YS (2011). Regulation of the Keap1/Nrf2 system by chemopreventive sulforaphane: implications of posttranslational modifications. *Annals of the New York Academy of Sciences*.

[8] Keum YS, Jeong WS, Kong AN (2004). Chemoprevention by isothiocyanates and their underlying molecular signaling mechanisms. *Mutation Research*.

[9] Itoh K, Chiba T, Takahashi S (1997). An Nrf2/small Maf heterodimer mediates the induction of phase II detoxifying enzyme genes through antioxidant response elements. *Biochemical and Biophysical Research Communications*.

[10] Kong AN, Owuor E, Yu R (2001). Induction of xenobiotic enzymes by the map kinase pathway and the antioxidant or electrophile response element (ARE/EpRE). *Drug Metabolism Reviews*.

[11] Kobayashi A, Kang MI, Okawa H (2004). Oxidative stress sensor Keap1 functions as an adaptor for Cul3-based E3 ligase to regulate proteasomal degradation of Nrf2. *Molecular and Cellular Biology*.

[12] Mitsuishi Y, Motohashi H, Yamamoto M (2012). The Keap1-Nrf2 system in cancers: stress response and anabolic metabolism. *Frontiers in Oncology*.

[13] Wakabayashi N, Itoh K, Wakabayashi J (2003). Keap1-null mutation leads to postnatal lethality due to constitutive Nrf2 activation. *Nature Genetics*.

[14] Kensler TW, Wakabayashi N (2010). Nrf2: friend or foe for chemoprevention?. *Carcinogenesis*.

[15] Nioi P, Nguyen T, Sherratt PJ, Pickett CB (2005). The carboxy-terminal Neh3 domain of Nrf2 is required for transcriptional activation. *Molecular and Cellular Biology*.

[16] McMahon M, Thomas N, Itoh K, Yamamoto M, Hayes JD (2004). Redox-regulated turnover of Nrf2 is determined by at least two separate protein domains, the redox-sensitive Neh2 degron and the redox-insensitive Neh6 degron. *The Journal of Biological Chemistry*.

[17] Itoh K, Wakabayashi N, Katoh Y (1999). Keap1 represses nuclear activation of antioxidant responsive elements by Nrf2 through binding to the amino-terminal Neh2 domain. *Genes and Development*.

[18] Uruno A, Motohashi H (2011). The Keap1-Nrf2 system as an in vivo sensor for electrophiles. *Nitric Oxide*.

[19] Ogura T, Tong KI, Mio K (2010). Keap1 is a forked-stem dimer structure with two large spheres enclosing the intervening, double glycine repeat, and C-terminal domains. *Proceedings of the National Academy of Sciences of the United States of America*.

[20] Dinkova-Kostova AT, Kostov RV (2012). Glucosinolates and isothiocyanates in health and disease. *Trends in Molecular Medicine*.

[21] Tong KI, Kobayashi A, Katsuoka F, Yamamoto M (2006). Two-site substrate recognition model for the Keap1-Nrf2 system: a hinge and latch mechanism. *Biological Chemistry*.

[22] Yu R, Chen C, Mo YY (2000). Activation of mitogen-activated protein kinase pathways induces antioxidant response element-mediated gene expression via a Nrf2-dependent mechanism. *The Journal of Biological Chemistry*.

[23] Kong AN, Yu R, Chen C, Mandlekar S, Primiano T (2000). Signal transduction events elicited by natural products: role of MAPK and caspase pathways in homeostatic response and induction of apoptosis. *Archives of Pharmacal Research*.

[24] Chen Z, Gibson TB, Robinson F (2001). MAP kinases. *Chemical Reviews*.

[25] Yu R, Lei W, Mandlekar S (1999). Role of a mitogen-activated protein kinase pathway in the induction of phase II detoxifying enzymes by chemicals. *The Journal of Biological Chemistry*.

[26] Keum YS, Owuor ED, Kim BR, Hu R, Kong AN (2003). Involvement of Nrf2 and JNK1 in the activation of antioxidant responsive element (ARE) by chemopreventive agent phenethyl isothiocyanate (PEITC). *Pharmaceutical Research*.

[27] Xu C, Shen G, Yuan X (2006). ERK and JNK signaling pathways are involved in the regulation of activator protein 1 and cell death elicited by three isothiocyanates in human prostate cancer PC-3 cells. *Carcinogenesis*.

[28] Lee KM, Kang K, Lee SB, Nho CW (2012). Nuclear factor-E2 (Nrf2) is regulated through the differential activation of ERK1/2 and PKC alpha/betaII by Gymnasterkoreayne B. *Cancer Letters*.

[29] Keum YS, Yu S, Chang PP (2006). Mechanism of action of sulforaphane: inhibition of p38 mitogen-activated protein kinase isoforms contributing to the induction of antioxidant response element-mediated heme oxygenase-1 in human hepatoma HepG2 cells. *Cancer Research*.

[30] Sun Z, Huang Z, Zhang DD (2009). Phosphorylation of Nrf2 at multiple sites by MAP kinases has a limited contribution in modulating the Nrf2-dependent antioxidant response. *PLoS ONE*.

[31] Salazar M, Rojo AI, Velasco D, de Sagarra RM, Cuadrado A (2006). Glycogen synthase kinase-3*β* inhibits the xenobiotic and antioxidant cell response by direct phosphorylation and nuclear exclusion of the transcription factor Nrf2. *The Journal of Biological Chemistry*.

[32] Rojo AI, Medina-Campos ON, Rada P (2012). Signaling pathways activated by the phytochemical nordihydroguaiaretic acid contribute to a Keap1-independent regulation of Nrf2 stability: role of glycogen synthase kinase-3. *Free Radical Biology and Medicine*.

[33] Rada P, Rojo AI, Chowdhry S, McMahon M, Hayes JD, Cuadrado A (2011). SCF/*β*-TrCP promotes glycogen synthase kinase 3-dependent degradation of the Nrf2 transcription factor in a Keap1-independent manner. *Molecular and Cellular Biology*.

[34] Jain AK, Jaiswal AK (2006). Phosphorylation of tyrosine 568 controls nuclear export of Nrf2. *The Journal of Biological Chemistry*.

[35] Jain AK, Jaiswal AK (2007). GSK-3*β* acts upstream of Fyn kinase in regulation of nuclear export and degradation of NF-E2 related factor 2. *The Journal of Biological Chemistry*.

[36] Huang HC, Nguyen T, Pickett CB (2000). Regulation of the antioxidant response element by protein kinase C-mediated phosphorylation of NF-E2-related factor 2. *Proceedings of the National Academy of Sciences of the United States of America*.

[37] Huang HC, Nguyen T, Pickett CB (2002). Phosphorylation of Nrf2 at Ser-40 by protein kinase C regulates antioxidant response element-mediated transcription. *The Journal of Biological Chemistry*.

[38] Cullinan SB, Zhang D, Hannink M, Arvisais E, Kaufman RJ, Diehl JA (2003). Nrf2 is a direct PERK substrate and effector of PERK-dependent cell survival. *Molecular and Cellular Biology*.

[39] Apopa PL, He X, Ma Q (2008). Phosphorylation of Nrf2 in the transcription activation domain by casein kinase 2 (CK2) is critical for the nuclear translocation and transcription activation function of Nrf2 in IMR-32 neuroblastoma cells. *Journal of Biochemical and Molecular Toxicology*.

[40] Mi L, Xiao Z, Veenstra TD, Chung FL (2011). Proteomic identification of binding targets of isothiocyanates: a perspective on techniques. *Journal of Proteomics*.

[41] Mi L, Wang X, Govind S (2007). The role of protein binding in induction of apoptosis by phenethyl isothiocyanate and sulforaphane in human non-small lung cancer cells. *Cancer Research*.

[42] Mi L, di Pasqua AJ, Chung FL (2011). Proteins as binding targets of isothiocyanates in cancer prevention. *Carcinogenesis*.

[43] Mi L, Hood BL, Stewart NA (2011). Identification of potential protein targets of isothiocyanates by proteomics. *Chemical Research in Toxicology*.

[44] Ouertatani-Sakouhi H, El-Turk F, Fauvet B (2009). A new class of isothiocyanate-based irreversible inhibitors of macrophage migration inhibitory factor. *Biochemistry*.

[45] Miseta A, Csutora P (2000). Relationship between the occurrence of cysteine in proteins and the complexity of organisms. *Molecular Biology and Evolution*.

[46] Marino SM, Gladyshev VN (2012). Analysis and functional prediction of reactive cysteine residues. *The Journal of Biological Chemistry*.

[47] Dinkova-Kostova AT, Holtzclaw WD, Cole RN (2002). Direct evidence that sulfhydryl groups of Keap1 are the sensors regulating induction of phase 2 enzymes that protect against carcinogens and oxidants. *Proceedings of the National Academy of Sciences of the United States of America*.

[48] Holland R, Fishbein JC (2010). Chemistry of the cysteine sensors in Kelch-like ECH-associated protein 1. *Antioxidants and Redox Signaling*.

[49] Takaya K, Suzuki T, Motohashi H, Onodera K, Satomi S, Kensler TW (2012). Validation of the multiple sensor mechanism of the Keap1-Nrf2 system. *Free Radical Biology and Medicine*.

[50] He X, Ma Q (2009). NRF2 cysteine residues are critical for oxidant/electrophile-sensing, Kelch-like ECH-associated protein-1-dependent ubiquitination-proteasomal degradation, and transcription activation. *Molecular Pharmacology*.

[51] Li W, Yu SW, Kong AN (2006). Nrf2 possesses a redox-sensitive nuclear exporting signal in the Neh5 transactivation domain. *The Journal of Biological Chemistry*.

[52] Li W, Kong AN (2009). Molecular mechanisms of Nrf2-mediated antioxidant response. *Molecular Carcinogenesis*.

[53] Li W, Jain MR, Chen C (2005). Nrf2 possesses a redox-insensitive nuclear export signal overlapping with the leucine zipper motif. *The Journal of Biological Chemistry*.

[54] Papp D, Lenti K, Modos D, Fazekas D, Dul Z, Turei D (2012). The NRF2-related interactome and regulome contain multifunctional proteins and fine-tuned autoregulatory loops. *FEBS Letters*.

